# β-catenin interaction with NHERF1 and RASSF1A methylation in metastatic colorectal cancer patients

**DOI:** 10.18632/oncotarget.12280

**Published:** 2016-09-27

**Authors:** Laura Schirosi, Annalisa Mazzotta, Giuseppina Opinto, Rosamaria Pinto, Giusi Graziano, Stefania Tommasi, Livia Fucci, Giovanni Simone, Anita Mangia

**Affiliations:** ^1^ Functional Biomorphology Laboratory, IRCCS Istituto Tumori “Giovanni Paolo II”, Bari, Italy; ^2^ Molecular Genetics Laboratory, IRCCS Istituto Tumori “Giovanni Paolo II”, Bari, Italy; ^3^ Scientific Direction, IRCCS Istituto Tumori “Giovanni Paolo II”, Bari, Italy; ^4^ Pathology Department, IRCCS Istituto Tumori “Giovanni Paolo II”, Bari, Italy

**Keywords:** β-catenin, NHERF1, RASSF1A, methylation, metastatic colorectal cancer, Pathology Section

## Abstract

There is an increasing need to identify new biomarkers in colorectal cancer (CRC) to further characterize this malignancy. β-catenin plays a central role in the Wnt signaling pathway. It also binds Na^+^/H^+^ exchanger regulating factor 1 (NHERF1) and interacts with the RAS-association domain family 1, isoform A (RASSF1A), but the mechanisms of this possible crosstalk are still not fully understood. In this study, we analyzed for the first time the different subcellular expression of β-catenin, NHERF1, and RASSF1A and their relationships with RASSF1A methylation in the progression of CRC. We assessed immunohistochemical expression and RASSF1A methylation in 51 patients with stage IV colorectal cancer. Biomarker expression analysis was carried out considering the tumor-adjacent normal tissue, the primary tumor, and the paired liver metastases. Regarding the tumor compartment, it was found that cytoplasmic β-catenin expression was positively correlated to membranous (*r* = 0.3002, *p* = 0.0323) and nuclear NHERF1 (*r* = 0.293, *p* = 0.0368). In the liver metastases, instead, we found a positive correlation of cytoplasmic and nuclear β-catenin expression with RASSF1A methylation (*r* = 0.4019, *p* = 0.0068 and *r* = 0.3194, *p* = 0.0345, respectively).

In conclusion, our results showed that β-catenin was the crucial protagonist in metastatic CRC through different effector proteins involved in this developing process. In tumor tissues, β-catenin was predominantly associated with NHERF1 in a dynamic context, while interestingly in liver metastases, we noted an increase of its oncogenic function through RASSF1A inactivation.

## INTRODUCTION

Colorectal cancer (CRC) is the third most frequent cancer type and its incidence continues to increase worldwide. There is therefore a need to identify new biomarkers to further characterize this malignancy [1]. It typically develops from a benign polyp, or adenoma, to malignant carcinoma and, in advanced stages, malignant cells metastasize to the lymph nodes or to distant organs. The liver is one of the most common sites of metastases [2]. In CRC, 90% of all tumors have a mutation in individual components of multiple oncogenes in the Wnt/β-catenin pathway [3] which is involved in various differentiation events during embryonic development and leads to tumor formation when aberrantly activated [4]. β-catenin is a 92-kDa cellular protein, which is associated with E-cadherin in maintaining cellular adhesion, and plays a primary role in Wnt signaling [5]. The different functions of the protein depend on the intracellular localization of the molecule; the membrane form is responsible for cell-to-cell adhesion, while the cytoplasmic protein is associated with Wnt signaling and is down regulated by the normal Adenomatous Polyposis Coli (APC) complex [5]. Mutations in components of the APC complex lead to increased levels of cytoplasmic β-catenin and its translocation to the nucleus [6]. Nuclear β-catenin acts as a transcription factor and binds to T-Cell Factor/Lymphoid Enhancer Factor (TCF/LEF), which leads to activation of target genes including *CyclinD1, c- Myc, CD44* and *Survivin,* and ultimately results in uncon­trolled cell proliferation in tumor cells [7]. Thus, nuclear β-catenin accumulation is a biomarker associated with invasion, metastasis and poor prognosis of CRC [8].

Shibata T *et al* [9]** have suggested a close association between β-catenin and Na^+^/H^+^ exchanger regulating factor 1/ezrin-radixin-moesin binding phosphoprotein of 50 kDa (NHERF1/EBP50) through its PDZ domain *in vitro* and *in vivo*. This complex enhances Wnt signaling and may cooperate in the development of cancer. NHERF1 is a 50-kDa adaptor protein composed of two tandem PDZ domains and a carboxyl (C)-terminal ERM-binding region, which is involved in the regulation of ion transporters and in the trafficking of many transmembrane molecules [10]. Hayashi *et al* [11] observed for the first time alterations of NHERF1 subcellular expression in CRC and have identified it as a new player in CRC progression. Our previous studies showed the expression and sub-localization of NHERF1 in different sites of metastatic CRC, suggesting a dynamic role of this protein in colon carcinogenesis [12, 13].

The Wnt/β-catenin and the Ras/mitogen-activated protein kinase (MAPK) pathways play important roles in cancer development. The MAPK pathway mediates cellular differentiation and proliferation. Both pathways have been studied discretely, but the mechanisms of possible crosstalk are still not fully understood. In CRC, mutations in KRAS and BRAF promote aberrant cell proliferation through the MAPK cascade [14, 15]. However, other KRAS downstream effectors are involved, some of which promote growth inhibitory effects, including differentiation, apoptosis, cell cycle arrest and senescence, but the mechanisms underlying such growth inhibitory actions remain poorly understood [16]. RAS-association domain family (RASSF) members belong to a recently identified family of putative tumor suppressor RAS effectors for which epigenetic silencing, by promoter methylation, occurred during cancer progression [17, 18]. RASSF1A is a member of this family, able to bind RAS in a GTP-dependent manner and to mediate the apoptotic effects of oncogenic RAS [19]. Methylation-associated inactivation of RASSF1A, described in many colorectal samples [20], suggests a synergistic effect between the silencing of the tumor suppressor gene RASSF1A and activation of the oncogene KRAS. Thus, RASSF1A methylation represents an alternative mechanism of aberrant RAS signaling [21]. In colon cancer, the interaction between RAS and β-catenin activation was studied [22, 23]. In detail, it was shown that RASSF1A inactivation, during intestinal tumor formation, may further deregulate Wnt pathway signaling leading to increased accumulation of nuclear β-catenin, contributing to the oncogenic effects of aberrant Wnt/β-catenin signaling [24].

In this study, we examined the expression and sub-localization of β-catenin, NHERF1 and RASSF1A proteins, in addition to the methylation status of RASSF1A, in tumor adjacent normal tissue, primary tumors, and paired liver metastases of metastatic CRC. The aim was to compare the levels of protein immunoreactivity and RASSF1A methylation in the progression of CRC. We studied, for the first time, the interactions and relationships among the three proteins and RASSF1A methylation in order to investigate their biological meaning in metastatic CRC.

## RESULTS

### β-catenin, NHERF1 and RASSF1A immunostaining in tumor-adjacent normal tissue, primary tumor and paired liver metastases of metastatic CRC

In the tumor-adjacent normal tissue (ANT) membranous β-catenin staining was noted with a median value of expression equal to 50% of positive cells (range 5-100 %), and only in 5 cases (9.8%) a cytoplasmic staining was also observed (range 0-50% and median value of 0%). Nuclear staining was completely absent. NHERF1, instead, showed a membranous, cytoplasmic and nuclear staining. In detail, NHERF1 membranous expression had a median value of 5% of positive cells (range 0-30%). The median value of cytoplasmic expression was 10% (range 0-60%), and 11% for nuclear localization (range 0-48%). In detail, considering cytoplasmic and nuclear immunoreactivity, 11 cases (21.6%) showed only nuclear staining, while in 4 cases (7.8%) only cytoplasmic staining was noted and 2 cases (3.9%) were entirely un-immunoreactive for both the localizations. In the ANT, RASSF1A had a heterogeneous cytoplasmic staining intensity and the expression was present with the same range of values in all samples (median 45% of positive cells, range 42-46%), thus it was not considered for the statistical analyses.

In the primary tumor (T) samples β-catenin expression was observed in the membranous localization with a median value of 75% of positive cells (range 0-90%), in the cytoplasmic compartment with a median value of 30% (range 0-85%), and in the nucleus with a range of expression between 0 and 50% of positive cells (median value 0%). It is noteworthy that 5/51 (9.8%) cases were completely negative for β-catenin immunoreactivity in all three different localizations. NHERF1 expression in T samples was observed in the membranous localization with a median value of 5% of positive cells (range 0-40%), while in the cytoplasm the median value of expression was 60% (range 20-80%), and it was expressed also in the nucleus with a median value of 18% (range 0-68%). In 42/51 (82.4%) cases cytoplasmic and nuclear immunostaining were observed in the same sample. RASSF1A expression was observed as granular staining with a median value of 30% of positive cells (range 0-100%) prevalently in the cytoplasmic compartment. Only 5 cases (9.8%) also showed a nuclear immunostaining and in 3 cases (5.9%) the immunoreactivity was only nuclear.

**Figure 1:**
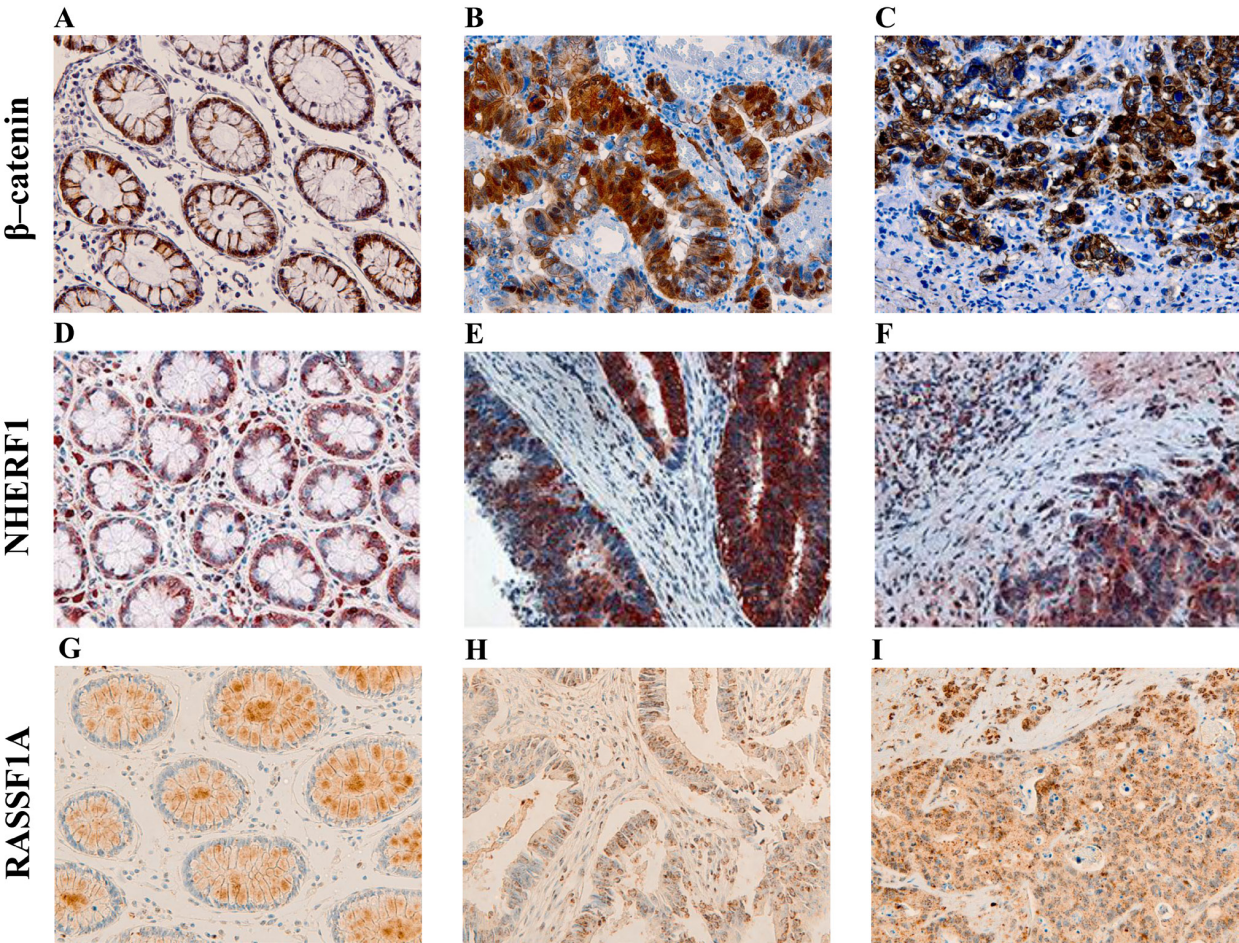
Representative images of the immunoreactivity of β-catenin, NHERF1 and RASSF1A Membrane and cytoplasmic localization of β-catenin in ANT **A.**, membrane, cytoplasmic and nuclear expression in T **B.**, membrane, cytoplasmic and nuclear β-catenin staining in LM **C.** NHERF1 immunoreactivity is present at the apical membrane, in cytoplasm and nucleus in ANT **D.**, while in T **E.** and LM **F.** it becomes mostly cytoplasmic and nuclear. Heterogeneous cytoplasmic staining intensity of RASSF1A in ANT **G.**, granular cytoplasmic staining in T **H.,** cytoplasmic and nuclear immunoreactivity in LM **I.** (original magnification x200).

In the paired liver metastases (LM) β-catenin immunoreactivity was observed in the membranous localization with a median value of 50% of positive cells (range 0-90%), in the cytoplasm with a range of expression between 0 and 60% (median value 0%) and in the nucleus with a range of expression between 0 and 28% (median value 0%). The same 5 patients who had T samples completely negative for β-catenin level also had negative LM, moreover there was another case with negative β-catenin expression in all three compartments. Membranous NHERF1 immunoreactivity was detected only in 3 cases (range 0-20%, median value of 0%), while the prevalent expression was noted in the cytoplasmic compartment, with a median value of 70% of positive cells (range 30-80%), and in the nucleus, with a median value of 20% (range 0-72%). In detail, cytoplasmic and nuclear labeling was observed in 40/51 cases (78.4%) in the same sample. For the other 4 patients we noted the complete loss of nuclear NHERF1 expression from T to LM. RASSF1A was expressed with a median value of 60% of positive cells (range 0-98%). The immunostaining was predominantly granular cytoplasmic but we noted 4/51 cases (7.8%) with only nuclear expression and 17/51 (33.3%) cases with both cytoplasmic and nuclear staining. Representative images of the immunoreactivity of β-catenin, NHERF1 and RASSF1A proteins in ANT, T and LM are shown in Figure 1 (A-I).

**Table 1:** Clinicopathological characteristics of 51 metastatic CRC patients

Characteristics	No. of patients
**Total series**	51
Age, median (range)	63 years (44-85)
**Gender**	
M	31
F	20
**Histology subtype**	
Adenocarcinoma	51
**Grade of differentiation**	
Low	12
High	39
**Status**	
T3	34
T4	17
**Nodal involvement**	
N1	14
N2, N3	37

### RASSF1A methylation analysis in metastatic CRC

The variation of RASSF1A promoter methylation was analyzed in T and in pair LM, highlighting a different frequency of inactivation between the two tumor compartments. In fact, RASSF1A appeared significantly more frequently methylated in LM than in T (85% *vs*. 35%, *p* = 0.015). Furthermore, over the frequency of RASSF1A promoter inhibition, we also evaluated the possible difference in methylation levels between the two sites. In detail, the RASSF1A methylation median level [interquartile range] was higher in LM than in the T compartment (15.33[3.03-48.69] and 0[0-0.67], respectively) in a statistically significant manner (*p* < 0.0001, by Wilcoxon signed-rank test).

### Expression analysis of β-catenin, NHERF1 and RASSF1A in metastatic CRC

**Figure 2:**
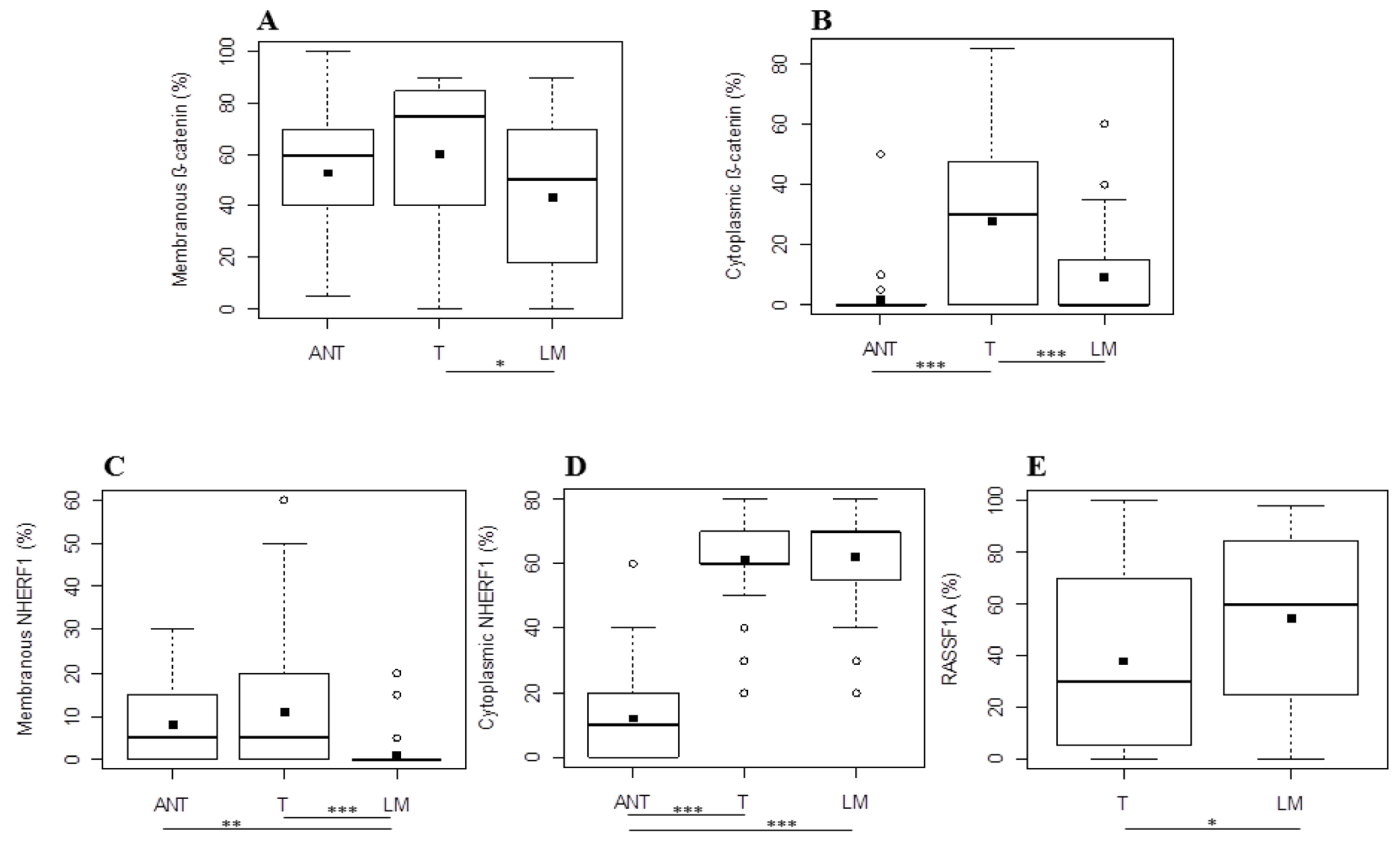
Protein expression analysis in metastatic CRC Differential expression levels of membranous **A.** and cytoplasmic **B.** β-catenin, membranous **C.** and cytoplasmic **D.** NHERF1 and RASSF1A **E**. among the different tissue compartments. Black square in each box represents the mean percentage of positive cells. Circle indicates outlier and horizontal line in each box indicates the median. Abbreviation: ANT, tumor- adjacent normal tissue; T, primary tumor; LM, liver metastasis. ***p < 0.0001; **p < 0.001; *p < 0.05.

Membranous β-catenin expression demonstrated a statistically significant difference (*p =* 0.0047, by the Kruskal-Wallis test) among the three compartments (ANT = 60[40-70], T = 75[40-85], LM = 50[18-70]) and in particular its median expression was higher in T than in LM (*p* = 0.0102, by Tukey's post-hoc analysis) (Figure 2A). The median cytoplasmic β-catenin expression, instead, was statistically higher in T than the ANT (*p* < 0.0001) and LM (*p* < 0.0001) compartment (Figure 2B) by Tukey's post-hoc test, with a significantly different expression (*p* < 0.0001, Kruskal-Wallis test) among ANT, T and LM (0[0-0], 30[0-50], 0[0-15], respectively). Protein analysis revealed also that membranous NHERF1 expression had a statistically significant difference (*p* < 0.0001, by the Kruskal-Wallis test) among the ANT, T and LM compartments (5[0-15], 5[0-20], 0[0-0], respectively), highlighting that its median expression was statistically higher in ANT than LM (*p* = 0.0008) and in T than LM (*p* < 0.0001), by Tukey's post-hoc test (Figure 2C). Moreover, cytoplasmic NHERF1 was differentially expressed among ANT, T and LM (10[0-20], 60[60-70] and 70[50-70] respectively) in a statistically significant manner (*p* < 0.0001, by Kruskal-Wallis test), and in particular Tukey's post-hoc test showed that the median expression of cytoplasmic NHERF1 in the ANT compartment was statistically lower than T (*p* < 0.0001) and LM (*p* < 0.0001) (Figure 2D). The Wilcoxon signed-rank test showed that the median expression of the RASSF1A protein in T was significantly lower that LM (30[2-70] and 60[25-85], respectively with *p* = 0.0256) (Figure 2E).

### Correlation analysis among expression of β-catenin, NHERF1 and RASSF1A and the methylation status of RASSF1A in metastatic CRC

**Figure 3:**
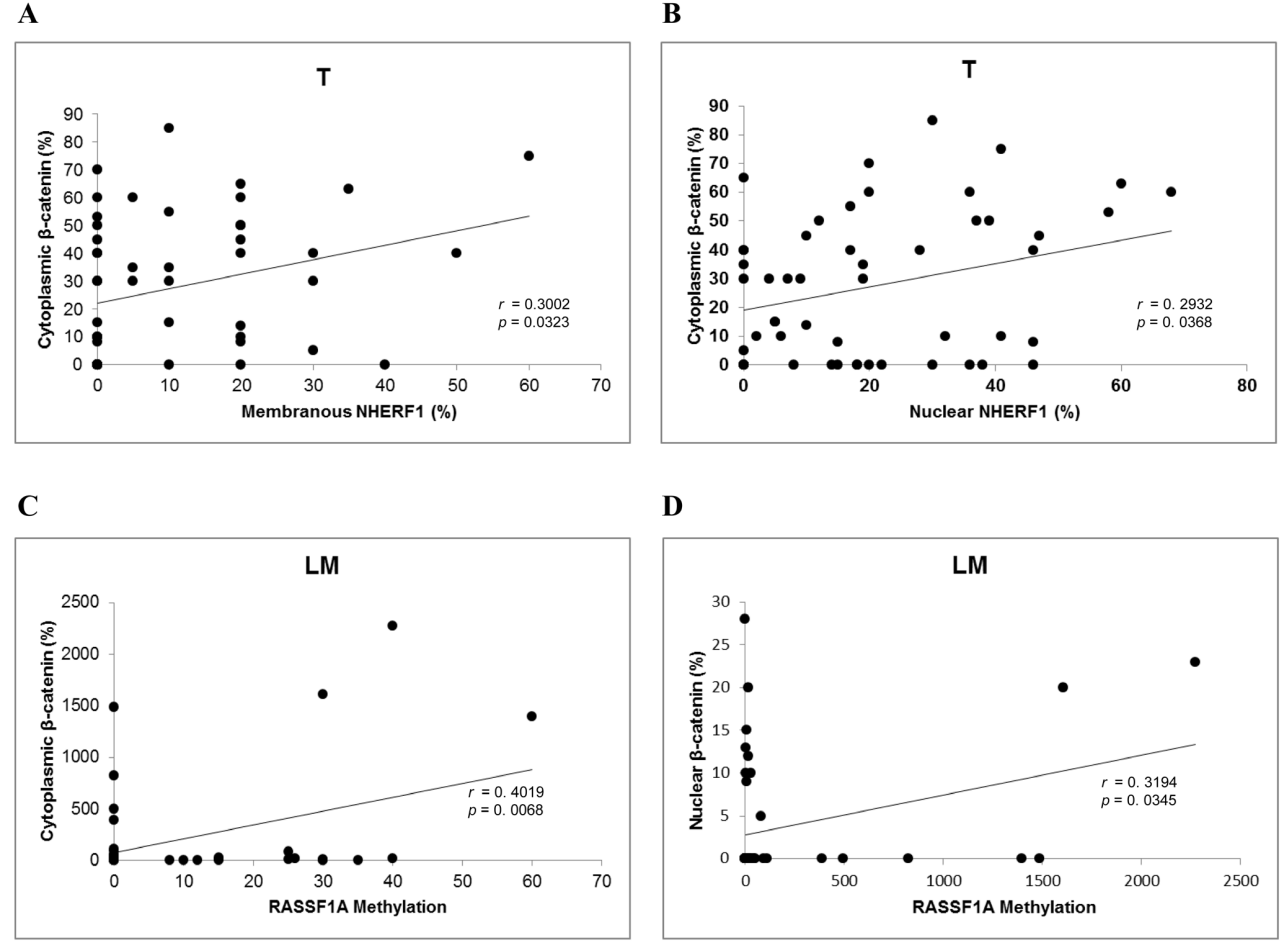
Correlation analysis among β-catenin, RASSF1A and NHERF1 in metastatic CRC In T samples cytoplasmic β-catenin correlated significantly with membranous **A.** and nuclear **B.** NHERF1, while in LM RASSF1A methylation correlated significantly with cytoplasmic **C.** and nuclear **D.** β-catenin. Abbreviation: T, primary tumor; LM, liver metastasis.

We performed correlation analyses for each compartment (ANT, T, LM), among the immunohistochemical expression of β-catenin, NHERF1, RASSF1A proteins in their different localizations where evaluated (membrane, cytoplasm and nucleus), and RASSF1A methylation. In the ANT compartment we did not obtain any statistically significant association. Regarding the T compartment, the Pearson rank test showed that cytoplasmic β-catenin expression was positively correlated to membranous NHERF1 (*r* = 0.3002, *p* = 0.0323) (Figure 3A) and nuclear NHERF1 expression (*r* = 0.293, *p* = 0.0368) (Figure 3B). Concerning LM, however, the Pearson rank test showed a positive correlation between cytoplasmic β-catenin expression and RASSF1A methylation (*r* = 0.4019, *p* = 0.0068) (Figure 3C). Moreover, also nuclear β-catenin expression was positively correlated to RASSF1A methylation (*r* = 0.3194, *p* = 0.0345) (Figure 3D).

The association analyses of the considered biomarkers in relation to the clinicopathological characteristics of the tumors did not show any statistically significant result.

## DISCUSSION

There is an increasing demand for biomarkers in colon cancer for risk assessment, early detection, prognosis, and surrogate end points. We know that β-catenin plays a central role in the Wnt signaling pathway in CRC, binding NHERF1 [25] and interacting with RASSF1A [26]. In this context, we observed the different localization and expression of β-catenin, NHERF1, and RASSF1A in primary CRC and paired liver metastases. To our knowledge, this is the first study to analyze the interactions and relationships among the three proteins, their localization and RASSF1A methylation.

Physiologic membrane β-catenin localization is responsible for cell-to-cell adhesion, but a cytoplasmic and nuclear expression when deregulation of Wnt signaling occurred has also been reported [4, 27]. Mechanisms of β-catenin shuttling between the membrane and cytoplasm or nucleus are only beginning to be understood [25]. In the present study, we observed a higher expression of cytoplasmic β-catenin in primary tumors than in the tumor-adjacent normal tissue, probably compatible with the onset of an aggressive phenotype. Moreover, we observed a higher expression of membranous and cytoplasmic β-catenin in the tumor than in paired liver metastases. This implies that β-catenin, in tumors, could have a physiologic role, but at the same time it could already assume also an oncogenic function by its cytoplasmic localization. In tumor and liver metastases tissues, β-catenin revealed heterogeneous sub-localizations, highlighting that different mechanisms, other than β-catenin or APC gene mutations, could control this shift [28]. β-catenin was shown to interact with NHERF1 through its PDZ2 motif *in vitro and in vivo* [9]. NHERF1 is physiologically localized mainly at the apical membrane in human epithelial tissue [29] but it was also found to be expressed in the cytoplasm and nucleus [12]. Hayashi *et al* [11] have observed changes in the expression and distribution of NHERF1 during CRC progression. Considering this aspect and in agreement with our previous results [12], in this study we observed membranous NHERF1 localization higher both in tumor-adjacent normal tissue and in tumors than in paired liver metastases. These results confirmed that NHERF1 membrane localization was required to maintain epithelial morphology in normal tissue and could suggest the same role also in primary tumors. On the contrary, in paired liver metastases this physiologic function was lost. Moreover, tumor samples showed a higher cytoplasmic NHERF1 expression than the tumor-adjacent normal tissues. In a similar way, Hayashi's model reported a cytoplasmic re-expression in CRC progression [11]. These results suggested that cytoplasmic NHERF1 expression could be needed to develop the oncogenic event in carcinoma and different mechanisms could probably control its subcellular sub-localization, such as gene mutations and phosphorylation [30]. We noted that membranous NHERF1 and β-catenin expression showed the same trend in primary tumors compared to the paired liver metastases. We could speculate that, also in the tumor, the physiologic role of NHERF1 was required to support β-catenin localization at the cell-cell junction [25]. At the same time, the shift of β-catenin and NHERF1 from the membrane to the cytoplasm, reported in tumor tissues, could be explained as it is linked to the oncogenic condition in tumor progression. These data are in agreement with previous studies which have shown a distinct distribution pattern of β-catenin and NHERF1, where their membranous and cytoplasmic expression was observed in the central mass of the tumor while nuclear localization was found at the invasive front [31, 32]. In addition, our results showed a significant correlation between cytoplasmic β-catenin and nuclear NHERF1 levels in tumor samples confirming the interaction between the two proteins and their potential oncogenic role also in CRC [9]. However, we found a significant positive correlation between cytoplasmic expression of β-catenin and also membranous NHERF1 expression. These results are representative of the dynamism of the two proteins which may probably depend on environmental signaling, their post-translational modifications and other interacting partners. This apparently discordant last finding could furthermore propose that, in CRC, the interaction between β-catenin and NHERF1 is not the only way by which β-catenin could switch its own localization from the membrane to the cytoplasm and nucleus to modulate transcriptional activity.

Interestingly, in liver metastases our results highlighted a different scenario compared to primary tumors. The poor survival of patients with CRC metastatic disease revealed the need for a better comprehension of the metastatic process. Probably, CRC metastases are genetically and epigenetically heterogeneous from the primary neoplasm, requiring a different approach and treatment. To clarify epigenetic changes occurring during CRC progression, in this study we proposed to investigate also RASSF1A methylation and its expression in primary tumors and in paired liver metastases. The RASSF1 gene frequently shows allelic loss in many types of solid tumor, and the RASSF1A, its major isoform, is a tumor suppressor frequently inactivated in cancer cells as a result of hypermethylation of a promoter CpG island [33]. RASSF1A silencing by promoter methylation has been shown also in CRC with frequencies from around 2% for adenomas to 60% for malignant stages [18, 34, 35].

However, there are very few studies about differences in RASSF1A methylation between primary CRC and metastatic samples. As already previously shown [36], also in this study we observed a higher frequency and level of methylation of RASSF1A in paired liver metastases than in primary tumors, which may explain its oncosuppressor function loss in paired liver metastases. In addition, our results demonstrated a positive correlation between RASSF1A methylation and nuclear β-catenin expression in these samples, highlighting a Wnt pathway activation mediated by RASSF1A in CRC progression. It had already been found that RASSF1A silencing brought about increased nuclear β-catenin accumulation, due to an inhibition of β-catenin degradation, in order to perform its oncogenic function [26].

However, in our liver metastases cohort, RASSF1A epigenetic inactivation resulted also significantly related to cytoplasmic β-catenin accumulation. To explain this association, we proposed in the metastatic samples a key role for β-catenin, whose translocation into the nucleus may lead to cell proliferation activation and apoptosis inhibition. In this study, we also found that the expression of RASSF1A in liver metastases was statistically higher than in tumors, and this finding was in apparent discordance with the increased methylation level in liver metastases. It is to note, however, that in the metastatic cohort compared to tumor samples we found a higher number of cases with cytoplasmic and contextually nuclear RASSF1A expression. Almost all of these cases were also methylated. We speculated that this expression could have a not entirely clear biological meaning, and for these reasons there was a lack of correlation with methylation status. In addition, in a previous study, Hu *et al* [37] similarly found no correlation between the expression of the RASSF1A protein and RASSF1A promoter methylation in hepatocellular carcinoma. We suggest, in agreement with these authors, that RASSF1A methylation is not the only mechanism to regulate protein expression in primary hepatocellular carcinoma, and probably also in liver metastases originating from different primary tumors.

In conclusion, our results showed a heterogeneous distribution of β-catenin and NHERF1, confirming a dynamic role of these two proteins in metastatic CRC. In tumor tissues, β-catenin functioned as an active protagonist, and it was predominantly associated with NHERF1, because the two proteins have the same membranous and cytoplasmic trend of expression. In paired liver metastases, interestingly, we noted an increase of the oncogenic role of β-catenin through RASSF1A inactivation. Thus β-catenin confirmed its crucial function in CRC progression through different effector proteins involved in this dynamic process.

Since our findings were limited by the small sample size, further studies could be needed to confirm these preliminary results.

## MATERIALS AND METHODS

### Patients and tissue specimens

Formalin-fixed paraffin-embedded (FFPE) samples and clinicopathological data were obtained from 51 patients with stage IV CRC who all underwent surgical treatment and pathological examination in the IRCCS Istituto Tumori “Giovanni Paolo II” between 2001 and 2008. Two experienced pathologists confirmed all the diagnoses histologically. Tumors were graded and classified according to the World Health Organization criteria [38]. Institutional Review Board approval for the use of human tissue in this study was given by the Research Ethics Committee of the IRCCS Istituto Tumori “Giovanni Paolo II”. For each sample of the 51 patients, the following tissues were considered: ANT and T present on the same section, and the paired LM. The clinicopathological characteristics of the 51 tumors analysed are summarized in Table 1.

### Immunohistochemistry

β-catenin, NHERF1 and RASSF1A protein expression patterns were examined on 4 μm FFPE sections of primary tumor and paired adjacent normal mucosa present on the same section, and liver metastases for each patients with stage IV CRC. Full-sections were stained by using two methods: (1) the manual procedure for the detection of β-catenin and NHERF1 proteins and (2) an automated procedure for RASSF1A protein expression.

For the manual staining method, FFPE tissue sections on Superfrost Plus slides were heated at 60°C for 60 min, deparaffinized with xylene and rehydrated through a graded ethanol series. Epitope antigen retrieval was carried out in 0.01 M citrate buffer (pH 6.0) at 98°C in a water bath for 30 min. After cooling, slides were incubated with 1% bovine serum albumin (BSA) in 1X phosphate-buffered saline (PBS) for 30 min to block non-specific protein binding. Primary antibodies against β-catenin (rabbit monoclonal anti-beta catenin antibody, E247, Abcam, Cambridge, UK; dilution 1:150) and NHERF1 (rabbit polyclonal EBP50 antibody, PA1-090, Affinity Bioreagents, Golden, CO; dilution 1:150) were applied at 4°C overnight. After endogenous peroxidase activity blocking for 10 min with 3% H_2_O_2_, the sections were incubated with anti-rabbit secondary antibody conjugated with peroxidase labeled polymer (EnVision™+ System-HRP, Dako, CA, USA) for 1 hour at room temperature according to the manufacturer's instructions. The products of the antigen-antibody reactions of the anti-β catenin and anti-EBP50 antibodies were visualized by incubating the sections in 3,3′-diaminobenzidine (Liquid DAB+ Substrate Chromogen System, Dako, CA, USA) for 10 min and in 3-amino-9-ethylcarbazole (AEC+ Substrate-Chromoge, Dako, CA, USA) for 8 min, respectively. Afterwards the slides were briefly counterstained with Mayer's haematoxylin (Bio-Optica, MI, Italy) and aqueously mounted.

For the automated staining method, testing was performed using the anti-RASSF1A antibody (mouse monoclonal anti-RASSF1a antibody, 3F3, Abcam, Cambridge, UK; dilution 1:30) on the Benchmark XT platform (BenchMark XT, Ventana Medical Systems, Tucson, AZ) with Cell Conditioning 1 for 80 min, pre-peroxidase inhibition and primary antibody incubation for 1 h at 37°C. The OptiView DAB IHC Detection Kit and OptiView Amplification Kit (Ventana Medical Systems) were used to detect RASSF1A protein expression. Tissues were counterstained with Haematoxylin II and Bluing Reagent for 12 min and 4 min, respectively. Samples were dehydrated by sequential washes through 70% ethanol, 96% ethanol, absolute ethanol cleared in xylene and then mounted.

Appropriate positive and negative controls were included in the proteins' immunohistochemical assessments. Colon cancer FFPE sections were used as positive internal controls. For negative control, the primary antibody was omitted and replaced by PBS 1X pH 7.6.

### Immunohistochemical assessment

The slides were evaluated by light microscopy. Staining evaluation was performed independently by two observers who were blinded to the clinicopathological parameters. All slides were independently reviewed twice with a consensus decision made in all cases of disagreement. Cases with a difference of less than 5% were considered concordant. In discordant cases, an agreement was reached after reading peer review. In order to score the stains, five representative tumor areas for each section were selected at x200 magnification and expressed as a percentage of positive cells/section. For β-catenin and NHERF1 proteins, the immunoreactivity was evaluated separately for the cell membrane, cytoplasmic and nuclear compartments, in each type of tissue considered (ANT, T and LM). Positivity in each compartment was defined by the percentage of cells stained positive from 0 to 100 throughout the whole tumor. RASSF1A immuno-expression was assessed as the percentage of labeled cytoplasmic cells and as mixed cytoplasmic/nuclear staining where it was present [39].

### Genomic DNA extraction and quantitative methyl-specific PCR (QMSP) analysis

After macrodissection, DNA was extracted by T and LM tissues containing more than 70% of cancer cells. Samples were digested with SDS/proteinase K over night at 56°C, and DNA was extracted with the QIAamp DNA FFPE Tissue kit (Qiagen, Valencia CA) according to the manufacturer's protocol. Concentrations were estimated with the ND-8000 Spectrophotometer (NanoDrop Technologies, Wilmington, DE).

Two micrograms of DNA extracted from T and LM specimens were subjected to bisulphite treatment and DNA purification using the Epitect Bisulfite kit (Qiagen, Valencia CA) according to the manufacturer's instructions. The modified DNA was used as a template for real-time fluorogenic MSP. Amplification reactions were carried out in triplicate in 20 μl that contained 3 μl bisulfite-modified DNA, 600 nM forward and reverse primers, 200 nM probe, 5 U of Platinum Taq polymerase (ThermoFisher Scientific), 200 mM each of dATP, dCTP, dGTP, dTTP, and 5.5 mM MgCl. Primers and probe of the gene of interest were designed to specifically amplify the bisulphite modified region containing the putative methylated CpGs, whereas the primer and probe for the reference gene (ACTB) were designed to specifically amplify a bisulphite modified region not containing CpGs. Amplifications were carried out using the following conditions: one step at 95°C for 3 min, 50 cycles at 95°C for 15 sec, and from 60 to 62°C for 1 min. Amplification reactions were carried out in 96-well plates on a 7000 Sequence detector (ThermoFisher Scientific). Each plate included patient DNA samples, positive (CpG Genome Universal Methylated DNA, a completely methylated DNA) and negative (normal leukocyte DNA or DNA from a known unmethylated cell line) controls, and multiple water blanks. Serial dilutions (90-0.009 ng) of CpG Genome Universal Methylated DNA were used to construct a calibration curve for the ACTB gene and for the gene of interest. The relative level of methylated DNA in each sample was determined as a ratio of the quantity mean of the target gene to the quantity mean of the ACTB and then multiplied by 1,000 for easier tabulation [(average value of triplicates of target gene/average value of triplicates of ACTB) x 1,000]. A series of 10 normal colon tissues were used as a calibrator and presented a QMSP level of the considered gene < 1. As a consequence, a gene was considered “methylated” when the QMSP level was ≥1.

### Statistical analyses

To perform all the statistical analyses in this study, we chose to analyze the continuous variables in order not to disperse any information nor introduce subjective elements. Comparisons of the expression levels of the analysed biomarkers among various tissue compartments were assessed with the Wilcoxon signed-rank test or Kruskal-Wallis test followed by Tukey's post-hoc analysis. The correlation between β-catenin, NHERF1 and RASSF1A expression and RASSF1A methylation was tested using Pearson's Correlation Coefficient (r). The association between clinicopathological characteristics and the proteins of interest were calculated with the Mann-Whitney test. The categorical variables have been defined according to the clinical standards, while for age we chosen the median value as cut off. Statistical significance was achieved at a p-value < 0.05. All the analyses were performed using the Statistical Package for Social Software (SPSS, version 17.0).
